# Anionic polymer coating for enhanced delivery of Cas9 mRNA and sgRNA nanoplexes†

**DOI:** 10.1039/d4bm01290a

**Published:** 2024-12-11

**Authors:** Siyu Chen, Simone Pinto Carneiro, Olivia M. Merkel

**Affiliations:** a Ludwig-Maximilians-University, Department of Pharmacy, Pharmaceutical Technology and Biopharmaceutics Butenandtstraße 5-13 Munich 81377 Germany siyu.chen@cup.uni-muenchen.de olivia.merkel@lmu.de

## Abstract

Polymeric carriers have long been recognized as some of the most effective and promising systems for nucleic acid delivery. In this study, we utilized an anionic di-block co-polymer, PEG-PLE, to enhance the performance of lipid-modified PEI (C14-PEI) nanoplexes for delivering Cas9 mRNA and sgRNA targeting KRAS G12S mutations in lung cancer cells. Our results demonstrated that PEG-PLE, when combined with C14-PEI at a weight-to-weight ratio of 0.2, produced nanoplexes with a size of approximately 140 nm, a polydispersity index (PDI) of 0.08, and a zeta potential of around −1 mV. The PEG-PLE/C14-PEI nanoplexes at this ratio were observed to be both non-cytotoxic and effective in encapsulating Cas9 mRNA and sgRNA. Confocal microscopy imaging revealed efficient endosomal escape and intracellular distribution of the RNAs. Uptake pathway inhibition studies indicated that the internalization of PEG-PLE/C14-PEI primarily involves scavenger receptors and clathrin-mediated endocytosis. Compared to C14-PEI formulations, PEG-PLE/C14-PEI demonstrated a significant increase in luciferase mRNA expression and gene editing efficiency, as confirmed by T7EI and ddPCR, in A549 cells. Sanger sequencing identified insertions and/or deletions around the PAM sequence, with a total of 69% indels observed. Post-transfection, the KRAS-ERK pathway was downregulated, resulting in significant increases in cell apoptosis and inhibition of cell migration. Taken together, this study reveals a new and promising formulation for CRISPR delivery as potential lung cancer treatment.

## Abstract 2

A novel PEG-PLE/C14-PEI nanoplex formulation for delivering Cas9 mRNA and sgRNA, targeting KRAS G12S mutations in lung cancer cells demonstrates high gene-editing efficiency and significantly enhanced cancer cell apoptosis and migration inhibition.

## Graphical abstract

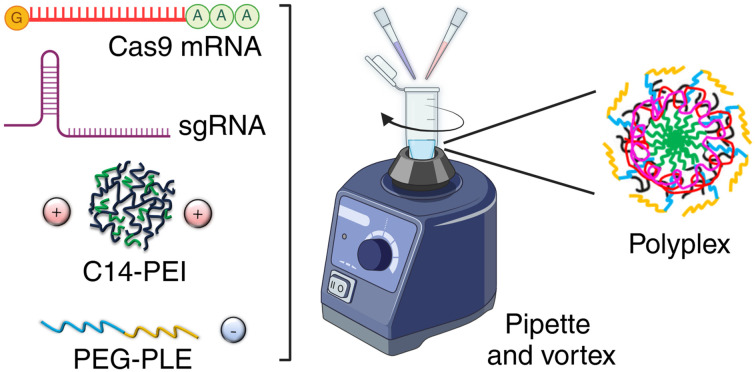

## Introduction

Clustered Regularly Interspaced Short Palindromic Repeats (CRISPRs) are sequences found in prokaryotic bacteria and archaea that function as part of an adaptive immune system. In 2012, Jennifer A. Doudna and Emmanuelle Charpentier introduced the CRISPR-Cas9 system as a groundbreaking tool for genome editing, marking a significant advancement in molecular biology.^1^ Their pioneering work earned them the Nobel Prize in Chemistry in 2020. Among the various CRISPR-Cas systems, Cas9 stands out as the most widely used and extensively studied. The mechanism by which CRISPR-Cas9 targets and edits DNA is closely tied to its structure. The system relies on the Cas9 protein, a 160-kilodalton endonuclease with a bi-lobed architecture, composed of the REC and NUC lobes.^2^ Cas9 forms a ribonucleoprotein complex with CRISPR RNA (crRNA) and *trans*-activating crRNA (tracrRNA), or a chimeric single-guide RNA (sgRNA), which guides it to the target double-stranded DNA (dsDNA).^3^ The sgRNA or crRNA-tracrRNA complex directs the Cas9 protein to cleave any DNA sequence that contains a 20-nucleotide complementary target sequence in vicinity to the protospacer adjacent motif (PAM) sequence. This two-component system can be easily used in applied science by designing the sgRNA to target virtually any DNA sequence in the genome, enabling precise site-specific double-strand breaks (DSBs). Once the DSB is introduced by Cas9, the cell can repair the break through two primary pathways: nonhomologous end joining (NHEJ) or homology-directed repair (HDR). NHEJ often results in small insertions or deletions (indels) at the cleavage site, while HDR allows for precise genome modification using a homologous repair template. Due to its efficiency, versatility, and relatively low cost, CRISPR-Cas9 has become a powerful and customizable tool for genome editing, offering advantages such as rapid onset, transient expression, and minimal off-target effects.^4^

CRISPR-Cas9 delivery methods typically include plasmid DNA,^5^ mRNA/sgRNA,^6^ and protein/sgRNA ribonucleoprotein complexes (RNPs).^7^ Over the past two decades, mRNA delivery technology has seen significant advancements. Most notably, the rapid development and widespread use of mRNA vaccines have played a crucial role in combating the COVID-19 pandemic.^8^ The success of nucleoside-modified mRNA-LNP vaccines developed by Moderna and Pfizer/BioNTech against SARS-CoV-2 marked a pivotal moment, establishing mRNA therapeutics as a viable approach in modern medicine. While mRNA vaccines have demonstrated the potential of mRNA delivery in nucleic acid therapy, the therapeutic applications of mRNA extend far beyond vaccines for infectious diseases. mRNA-based CRISPR-Cas9 therapeutics offer several distinct advantages.^9,10^ One key benefit is the ability to achieve transient expression, providing controlled and time-limited therapeutic effects.^11^ This feature reduces the risk of off-target effects, enabling more precise and safer delivery. Additionally, mRNA-based systems avoid the risk of genomic integration, thereby preserving the integrity of the host genome.^12^ Combined with their lower immunogenicity compared to viral vectors, these factors underscore the safety and growing interest in mRNA-based CRISPR-Cas9 delivery. Despite these advantages, effective delivery of mRNA *in vivo* and *in vitro* remains a significant challenge, limiting the full potential of CRISPR-mediated gene editing.^12^ Various strategies are being explored to address this challenge, including viral delivery,^13^ cell-penetrating peptides (CPPs),^14^ gold nanoparticles (AuNPs),^15^ lipid nanoparticles (LNPs),^16^ and polymeric carriers.^17^ Among these, polymer-based delivery systems, though often overlooked in favor of lipid nanoparticles, offer unique benefits. They allow for precise tuning of chemical properties to enhance mRNA protection, favorable pharmacokinetics, and targeted delivery.^18^ One of the most commonly used cationic polymers for nucleic acid delivery is polyethylenimine (PEI).^19^ PEI is known for its high loading capacity, efficient cellular internalization, strong endosomal disruption, and low cost.^18^ However, its strong cationic nature also poses challenges, including toxicity, which can lead to necrosis, apoptosis, and inflammation.^20^ In the context of mRNA delivery, PEI tends to have relatively low transfection efficiency because its strong binding to RNA can impair mRNA release from the complex.^21^ Therefore, optimizing PEI-based vehicles is essential for achieving safe and efficient mRNA delivery.

In previous studies, lipid-modified PEI has been proven successful for nucleic acid delivery.^22^ In our earlier study,^23^ we developed a lipid-modified PEI (C14-PEI) polyplex system to co-deliver Cas9 mRNA and sgRNA, achieving promising gene editing results in A549 lung cancer cells. However, this delivery system was characterized by large particle sizes and highly positive zeta potentials, which may limit its effectiveness and cause unwanted inflammatory effects *in vivo*. However, the co-delivery of Cas9 mRNA and sgRNA still faces challenges due to the necessity of considering multiple components.^24^ To enhance the biophysical and chemical properties of nanoparticles for *in vivo* applications, non-cationic polymers are often employed as core or shell stabilizers for mRNA and for positively charged segments.^19^ These polymers contribute to surface adsorption or charge shielding, improving the nanoparticles’ performance. Among these, poly(ethylene glycol) (PEG) is widely recognized for its role in drug delivery.^25^ PEG is highly hydrophilic and electrostatically neutral, and when present on the surface of nanoparticles, it provides colloidal stability through steric repulsion, which increases with the length of the PEG chains.^26,27^ Modifying PEI-based nanoparticles with PEG terminal groups has shown promise in targeting lung tissues, though this modification reduces stability against heparin compared to unmodified PEI polyplexes.^28^ Additionally, negatively charged macromolecules can serve as protective shells, shielding the positively charged nanoparticles and prolonging their circulation time in the bloodstream.^29–31^ For instance, anionic polysaccharides can either covalently bond with cationic materials or incorporate directly into nucleic acid complexes *via* electrostatic interactions, effectively masking the cationic regions of the delivery carriers.^25^ These findings underscore the significant role that PEG chains with anionic groups play in the performance of polyplexes. They also highlight the need for further research to fully understand how non-cationic block co-polymers influence mRNA delivery in CRISPR-Cas9 gene editing applications.

In this study, we utilized methoxy-poly(ethylene glycol)-*block*-poly(l-glutamic acid sodium salt) (PEG-*b*-PLE) as an auxiliary component in a lipid-modified PEI (C14-PEI) delivery system to target mutated *KRAS* with Cas9 mRNA and sgRNA. We systematically characterized and compared the C14-PEI and PEG-PLE/C14-PEI nanoparticles in terms of size, zeta potential, cytotoxicity, and encapsulation efficiency. The duration and degradation of the Cas9 mRNA and sgRNA were monitored using colocalization techniques under confocal laser microscopy. We also investigated the cellular uptake pathways and endosomal entrapment, followed by an assessment of luciferase mRNA expression and gene editing efficiency in A549 lung cancer cells. To evaluate the therapeutic relevance, we performed western blot analysis, wound healing assays, and cell apoptosis tests. Our findings showcase the innovative potential of PEG-PLE and C14-PEI blends as a robust delivery platform for mRNA delivery and gene editing. This unique combination introduces specific functional advantages by shielding particle surface charges in Cas9 mRNA and sgRNA delivery, including optimized size and zeta potential, enhanced stability, improved cellular uptake, successful mRNA expression, and efficient gene editing. In summary, this study underscores the evolving role of polymeric nanoparticles in advancing mRNA-based therapies, offering more possibilities for polymer-based CRISPR delivery systems aimed at targeting oncogenic mutations such as *KRAS* in cancer treatment.

## Experimental

### Materials

Methoxy-poly(ethylene glycol) (5000 Da)-*block*-poly(l-glutamic acid sodium salt) (7500 Da) (PEG-*b*-PLE) was obtained from Alamanda Polymers (Huntsville, AL, US). 1,2-epoxytetradecane, Branched PEI 600 Da, 4-(2-hydroxyethyl)-1-piperazineethanesul-fonic acid (HEPES), Dulbecco's Phosphate Buffered Saline (PBS), 0.05% trypsin-EDTA, Tris-buffered saline, Tween 20, RPMI-1640, fetal bovine serum (FBS), penicillin–streptomycin solution, 6-diamidino-2-phenylindole dihydrochloride (DAPI), skim milk, paraformaldehyde (PFA), agarose powder, and Cell Counting Kit-8 were purchased from Sigma-Aldrich (Darmstadt, Germany). SYBR™ Gold Stain, SYBR Safe DNA Gel Stain, Lipofectamine™ 2000, LysoTracker™ Green DND-26, Annexin V-AF488, GeneArt™ Genomic Cleavage Detection Kit, Phusion Hot Start II High-Fidelity PCR Mastermix, ExoSAP-IT™ Express PCR Product Cleanup Reagent, Pierce™ BCA Protein Assay kit, Novex™ WedgeWell™ 8–16% Tris-Glycin gel, Pierce™ Protease Inhibitor Tablets, RIPA buffer, SuperSignal™ West Femto Maximum Sensitivity Substrat were bought from Thermo Fisher Scientific (Planegg, Germany). ddPCR NHEJ Gene Edit Assay (primers and probes), ddPCR Supermix for Probes (no dUTP), cartridges, gaskets, droplet generation oil, and droplet reader oil were purchased from Bio-Rad, US. RNeasy Mini Kit (QIAGEN, US), pCp-AF488 (Jena Bioscience, Germany), Oligo Clean & Concentrator Columns (Zymo, Germany), Luciferase mRNA (RiboPro, Niederland), CleanCap® Cas9 mRNA (5moU) (Trilink Biotechnologies, US), cOmplete™ EDTA-free Protease Inhibitor Cocktail (Roche, Germany), Rotiphorese®NF 10× TBE Buffer (Carl Roth, Germany), propidium iodide (PI) (BD Biosciences, US), DNeasy Blood & Tissue Kit (Qiagen, US), and Amersham™ Protran® western blotting nitrocellulose membranes (Cytiva technologies, Germany) were obtained from the suppliers indicated. Methanol, ethanol, and acetone were provided by Ludwig-Maximilians-University Munich. The primary antibodies for p44/42 MAPK (Erk1/2) and Phospho-p44/42 MAPK (T202/Y204) were from Cell Signaling Technology (Danvers, MA, US). KRAS polyclonal antibody, Histone-H3 polyclonal antibody, and HRP-conjugated affinipure goat anti-rabbit IgG (H + L) secondary antibody are from Proteintech (Planegg, Germany). Cy5-mRNA was synthesized and labeled in the laboratory. sgRNA (*KRAS* G12S: 5′-CUUGUGGUAGUUGGAGCUAG-3′) was synthesized by Sigma-Aldrich. Primers for PCR (F: TTTGAGAGCCTTTAGCCGC, R: TCTACCCTCTCACGAAACTC) and primers for Sanger sequencing (F: TCTTAAGCGTCGATGGAG, R: ACAGAGAGTGAACATCATGG) were synthesized by Sigma-Aldrich (Darmstadt, Germany).

### C14-PEI synthesis

C14-PEI was synthesized by reacting 1,2-epoxytetradecane with branched PEI 600 Dalton through a ring cleavage reaction as in the previous report.^22^ Briefly, 1,2-epoxytetradecane and bPEI 600 Da were heated at 95 °C in absolute ethanol for 72 h while stirring. The product was then dialyzed with a 1000 Da cutoff in absolute ethanol, followed by ethanol removal using high-pressure nitrogen air.

### Nanoparticle preparation

C14-PEI nanoparticles were prepared by pipette mixing through electrostatic interactions. Specifically, 500 ng of luciferase mRNA or Cas9 mRNA with sgRNA at a molar ratio of 1 : 10 was added into 100 μL of 10 mM HEPES buffer, pH 7.4, and C14-PEI solution with an eightfold weight excess in comparison to total RNA (w/w 8) were added and mixed with RNA by pipetting and vortexing in HEPES buffer. The mixture was then incubated at room temperature for 1 hour. For PEG-PLE/C14-PEI nanoparticles, a predetermined amount of PEG-PLE was added to the RNA solution in the first step, which was then mixed with C14-PEI in HEPES buffer. The morphology of the polyplexes was examined using cryo-electron microscopy (Cryo-EM).

### Nanoparticle characterization

The size, polydispersity indices (PDI), and zeta (ζ) potential of nanoparticles were characterized using a Zetasizer Ultra (Malvern, UK). The nanoparticle suspension was added to a disposable micro-cuvette, and the hydrodynamic diameter and PDI were measured three times per sample using dynamic light scattering (DLS) at a 173° backscatter angle. Subsequently, the same suspension was transferred to a folded capillary cell for each sample to determine the zeta potential in triplicate using laser Doppler anemometry (LDA), with each run consisting of up to 100 scans. Results are presented as mean ± standard deviation (SD, *n* = 3).

Nanoparticle Tracking Analysis (NTA) combines light scattering and Brownian motion to determine the size distribution of nanoplexes in liquid suspension. By tracking individual particles’ mean squared displacement, the NTA software calculates their hydrodynamic diameter using the Stokes–Einstein equation.^32^ Using the NanoSight Pro system (Malvern Instruments, Amesbury, UK), subtle changes in particle population characteristics are detected, with real-time visual validation. For measurement, nanoplexes were vortexed and diluted in particle-free HEPES buffer to achieve a concentration within the recommended range (1 × 10^6^–1 × 10^9^ particles per mL). Videos were captured using the NanoSight NTA software version 3.4 in script control mode (3 videos, each 60 s) at 25 °C, with a syringe pump speed of 20. Each video consisted of 1500 frames, and camera levels were adjusted according to the scatter properties of the first measurement. Video analysis settings were fine-tuned by increasing the screen gain and adjusting the detection threshold for optimal single-particle tracking, while other parameters were set to default or automated.

### SYBR gold assay

To evaluate the mRNA encapsulation capacity of PEG-PLE/C14-PEI nanoparticles, SYBR Gold assays were conducted. SYBR Gold is a cyanine dye that binds to nucleic acids and exhibits fluorescence upon excitation. Briefly, nanoparticles were prepared as described earlier at weight-to-weight (w/w) ratio of 8 and with addition of PEG-PLE to C14-PEI at weight-to-weight (w/w) ratios of 0, 0.1, 0.2, 0.3, 0.4, and 0.5. Subsequently, 100 μL of each polyplex solution was added to black FluoroNunc 96-well plates (Fisher Scientific, Germany). A 4× SYBR Gold aqueous solution (30 μL per well) was then added to each well and incubated for 10 min in the dark. The fluorescence intensity was measured using a fluorescence plate reader (TECAN, Switzerland) with excitation at 485/20 nm and emission at 535/20 nm. The fluorescence intensity of free mRNA (polymer to RNA w/w = 0) was used as a control and set as 100% fluorescence.

### Agarose gel electrophoresis

Agarose gel electrophoresis was used to confirm the co-encapsulation of Cas9 mRNA and sgRNA and to perform the T7 endonuclease I (T7EI) assay. For each run, a 1% agarose gel, containing SYBR Safe dye at a 1 : 100 000 dilution, was prepared in Tris Borate EDTA (TBE) buffer. The nanoparticle samples, free RNA, and products from the T7EI assay were mixed with 6× DNA loading dye and then loaded onto the gel. Electrophoresis was carried out at 150 V for 40 min, and the gel was visualized using the ChemiDoc imaging system (Bio-Rad, US).

### Cell culture

A549 cells were cultured in complete RPMI-1640 medium supplemented with 10% heat-inactivated fetal bovine serum (FBS) and 1% penicillin–streptomycin. All cells were subcultured, maintained, and grown in an incubator at 37 °C in humidified air with 5% CO_2_.

### Cytotoxicity test

The cytotoxicity of nanoparticles was assessed using a CCK-8 assay in A549 cells. Specifically, 10 000 cells per well were seeded 24 h prior in a transparent 96-well plate (Fisher Scientific, Hampton, NH, USA). PEG-PLE/C14-PEI nanoparticles were freshly prepared at polymer w/w 0.5 and w/w 0.2. C14-PEI nanoplexes were used as a control. After removing the old medium, the fresh medium containing nanoparticles with different concentrations (1×, 5×, 10×, 20×, 40×, and 80× fold increase of PEG-PLE/C14-PEI w/w 0.2) was added to each well and incubated for 24 h at 37 °C and 5% CO_2_. Subsequently, the medium was aspirated, and a fresh medium containing CCK-8 solution (10 μL CCK-8 in 100 μL RPMI-1640 media) was added to each well. After incubating for 4 h, a water-soluble orange formazan product formed in the medium, and absorbance was measured at 450 nm using a Tecan plate reader. The experiment was conducted in triplicate, and the results are presented as mean values (*n* = 3), normalized to the percentage of viable cells relative to untreated cells (100% viability).

### Uptake pathway

To investigate the route of nanoparticle uptake, experiments with different types of specific uptake inhibitors were performed.^33^ A549 cells (100 000 per well) seeded 24 h prior to the experiment were incubated with nystatin (20 μg mL^−1^), dextran sulfate (100 μg mL^−1^), chlorpromazine (5 μg mL^−1^), or methyl-beta-cyclodextrin (1 mg mL^−1^) for 1 h followed by incubation with C14-PEI or PEG-PLE/C14-PEI nanoparticles containing Cy5-labeled mRNA for 2 h. Incubation at 4 °C for energy inhibition was set as a control. Positive control cells without inhibitor treatment were transfected with polyplexes, and untreated cells served as a blank control. After 2 h of transfection, the cells were washed with PBS and detached using 0.05% trypsin-EDTA. The detached cells were then collected in 1.5 mL Eppendorf tubes and centrifuged at 300 g for 5 min. After centrifugation, the supernatant was aspirated, and the cells were washed again with PBS, followed by a second centrifugation step. The resulting cell pellet was resuspended in fresh PBS, and the fluorescence intensity was measured using an Attune NxT flow cytometer (Thermo Fisher, Planegg, Germany) with excitation at 651 nm and emission at 670 nm. The experiments were performed in triplicate. Results are shown as a percentage of median fluorescence intensity normalized to not inhibited positive control samples (100%).

### Endosomal entrapment

To visualize the endosomal entrapment of nanoplexes, A549 cells were imaged using confocal laser scanning microscopy (CLSM, Leica SP8 inverted; software: LAS X, Leica Microsystems GmbH, Wetzlar, Germany) following transfection with fluorescently labeled mRNA. A total of 10 000 A549 cells were seeded into ibiTreat μ-Slide 8-well plates (ibidi, Gräfelfing, Germany) and transfected with PEG-PLE/C14-PEI (w/w 0.5) containing Cy5-mRNA. Lipofectamine 2000, PEI nanoparticles, and free Cy5-mRNA were used as controls. After incubation at 37 °C with 5% CO_2_ for 4, 8, or 24 h, the cells were stained with LysoTracker Green DND-26 in pre-warmed cell culture medium for 1 hour. Following medium removal, cells were washed and fixed with 4% paraformaldehyde (PFA) for 15 min in the dark and then washed again with PBS. DAPI was added to the appropriate wells at a final concentration of 1 μg mL^−1^ in PBS and incubated for 20 min at room temperature in the dark. After washing, the cells were maintained in PBS at 4 °C for subsequent analysis by CLSM. For imaging, excitation was achieved using a diode laser at 405 nm, an argon laser at 488 nm, and a helium–neon laser at 650 nm. Emission was recorded in the blue channel (420–480 nm) for DAPI, the green channel (500–550 nm) for LysoTracker Green, and the red channel (650–720 nm) for Cy5-mRNA fluorescence.

### Co-localization of mRNA and sgRNA

To assess the duration and degradation of Cas9 mRNA and sgRNA within cells using PEG-PLE/C14-PEI nanoparticles, we employed co-localization techniques with CLSM. Cas9 mRNA and sgRNA were labeled with Cy5 and AF488, respectively, for visualization. Cas9 mRNA was synthesized through *in vitro* transcription (IVT) using a mixture of nucleoside triphosphosphates (NTPs) containing Cy5-UTP (Jena Bioscience, Germany). The linearized DNA templates, NTP mixture, Cy5-UTP, and T7 polymerase were combined according to the HiScribe® T7 ARCA mRNA Kit with tailing (NEB, US) protocol. The reaction was incubated overnight at 37 °C, and the RNA products were purified using the RNeasy Mini Kit (Qiagen, Hilden, Germany) and verified by agarose gel electrophoresis. For sgRNA labeling, pCp-AF488 (Jena Bioscience, Germany) was added at the 3′ end. The reaction mixture included sgRNA, pCp-AF488, ATP, T4 RNA Ligase, Reaction Buffer, RNAse inhibitor, 10% DMSO, and 15% PEG8000, which was incubated for 18 h at 16 °C. The AF488-labeled sgRNA was purified from the reaction mix using Oligo Clean & Concentrator Columns (Zymo, Germany) and analyzed by UV/VIS spectroscopy (A260 nm: total RNA population; A494 nm: AF488-labeled RNA). A549 cells were transfected with PEG-PLE/C14-PEI nanoparticles containing Cy5-labeled Cas9 mRNA and AF488-labeled sgRNA. At various time points (1 h, 4 h, 8 h, 24 h, 36 h, and 48 h), cells were fixed with 4% paraformaldehyde (PFA) and nuclei were stained with DAPI. Images were captured using CLSM and analyzed with ImageJ to determine the duration and degradation of the mRNA and sgRNA.

### Luciferase mRNA expression

To evaluate the translational efficiency of mRNA delivered by PEG-PLE/C14-PEI, we quantified the expression of the luciferase protein reporter mRNA (Fluc mRNA) using a plate reader (TECAN, Männedorf, Switzerland). A549 cells were seeded at a density of 10 000 cells per well in 96-well plates containing 200 μL of growth medium. Following incubation in a cell culture incubator (37 °C, 5% CO_2_) for 24 h, the cells were transfected with PEG-PLE/C14-PEI nanoparticles encapsulating Fluc mRNA at w/w 0, w/w 0.1, w/w 0.2, w/w 0.3, w/w 0.4, w/w 0.5. PEI served as a control treatment. After 24 h of transfection, cells were washed with PBS and lysed by lysis buffer followed by incubation at room temperature for 30 min. Of each sample, 35 μL lysate was added to a white 96-well plate, and the samples were activated by 0.25 mM luciferin substrate with an autosampler (TECAN, Männedorf, Switzerland). Subsequently, the samples were measured for relative light unit (RLU) of luminescence with the plate reader. Results are presented as mean ± standard deviation (SD, *n* = 3).

### T7 endonuclease I (T7EI) assay

The T7EI assay was conducted according to the manufacturer's protocol using the GeneArt™ Genomic Cleavage Detection Kit. A549 cells were initially seeded in 6-well plates at a density of 100 000 cells per well in 1.5 mL of medium 24 h before the experiment. Following a media change, cells were transfected with PEG-PLE/C14-PEI nanoparticles containing Cas9 mRNA and sgRNA. Lipofectamine 2000, PEI, and C14-PEI were included as controls. Transfected cells were then incubated at 37 °C with 5% CO_2_ for 48 h. Subsequently, cells were washed with PBS, harvested using 0.05% trypsin-EDTA, and collected by centrifugation into 1.5 mL Eppendorf tubes. The cell pellets were lysed using lysis buffer, and the resulting lysates were utilized for polymerase chain reaction (PCR) amplification of sequences containing *KRAS* alleles. Following PCR amplification, the PCR products underwent re-annealing and treatment with the detection enzyme as per the kit's instructions. The positive control sample provided in the kit was included for validation. Agarose gel electrophoresis was performed to visualize the cleavage products, and images were captured using the ChemiDoc imaging system as described in the section of agarose gel electrophoresis. Data analysis was conducted using Image Lab Software (Bio-Rad, USA).

### Droplet digital PCR

A549 cells were transfected in 6-well plates using PEG-PLE/C14-PEI nanoparticles with Cas9 mRNA and sgRNA for 48 h, with Lipofectamine 2000, PEI, and C14-PEI used as controls. Genomic DNA was extracted from both untreated and treated A549 cells using the DNeasy Blood & Tissue Kit (Qiagen), and the DNA concentration was quantified using a Nanodrop spectrophotometer. Primers and probes were custom-designed and obtained from Bio-Rad (Feldkirchen, Germany). The reaction mixtures for droplet digital PCR (ddPCR) contained 2× ddPCR Supermix for Probes (no dUTP), with final concentrations of 900 nM for each primer and 250 nM for each FAM- or HEX-labeled probe. A total of 100 ng of template DNA was added to achieve a final reaction volume of 20 μL. Standard Bio-Rad reagents and consumables, including cartridges, gaskets, droplet generation oil, and droplet reader oil, were used. After droplet generation, droplets were carefully transferred to a 96-well PCR plate and sealed using the PX1 PCR Plate Sealer (Bio-Rad). The PCR conditions were as follows: initial denaturation at 95 °C for 10 min, followed by 40 cycles of denaturation at 94 °C for 30 s, annealing/extension at 55 °C for 3 min, and a final extension step at 98 °C for 10 min, followed by a hold at 4 °C. The ramp rate was set at 2 °C s^−1^. Droplets were read using the QX200 Droplet Reader (Bio-Rad), and each reaction included a no-template control (NTC). Data analysis was performed using QuantaSoft Software.^34^

### Sanger sequencing

Genomic DNA was extracted from A549 cells 48 h post-transfection with PEG-PLE/C14-PEI nanoparticles using the DNeasy Blood & Tissue Kit. To visualize the gene sequence after gene editing, PCR was performed using a pair of primers designed to target regions before and after the cleavage site, yielding a PCR product of approximately 500 base pairs. The Phusion Hot Start II High-Fidelity PCR Mastermix was utilized for PCR amplification. The cycling conditions were as follows: initial denaturation at 98 °C for 30 s, followed by 35 cycles of denaturation at 98 °C for 10 s, annealing at 61.5 °C for 30 s, extension at 72 °C for 30 s, and a final extension at 72 °C for 10 min. PCR products were verified by electrophoresis on 1% agarose gels. Following gel verification, PCR products were purified using the ExoSAP-IT™ Express PCR Product Cleanup Reagent. The purified PCR products were subsequently used for Sanger sequencing to determine the sequence changes resulting from the gene editing process. The results were analyzed by the ICE CRISPR analysis tool.^35^

### Western blot

To assess the ability of PEG-PLE/C14-PEI nanoparticles to inhibit downstream signals in the KRAS pathway, A549 cells were seeded in 6-well plates and allowed to grow for 24 h to reach a density of 1 × 10^5^ cells per well. The cells were then treated with PEG-PLE/C14-PEI nanoparticles and incubated at 37 °C with 5% CO_2_ in a humidified incubator for 48 h. Following treatment, cells were washed with ice-cold PBS and lysed in RIPA buffer containing phosphatase inhibitors and protease inhibitors. The protein content in the lysates was quantified using the Pierce™ BCA Protein Assay kit (Thermo Fisher), and equal amounts of protein were loaded onto SDS-PAGE (Novex™ WedgeWell™ 8–16% Tris-Glycin gel). Separated proteins were then transferred onto nitrocellulose membranes, which were subsequently blocked with 5% skim milk in TBST (Tris-buffered saline with 1% Tween 20) for 1 hour at room temperature. Membranes were then incubated overnight at 4 °C with primary antibodies targeting specific proteins of interest in the KRAS pathway. After primary antibody incubation, membranes were washed three times with 1% TBST and then incubated with horseradish peroxidase (HRP)-conjugated secondary antibodies at room temperature for 1 hour. Protein bands were visualized using chemiluminescence substrates and imaged immediately using the ChemiDoc imaging system (BioRad). Between antibody stainings, membranes were treated with stripping buffer for 30 min to remove bound antibodies, followed by washing with TBST and re-blocking with 5% skim milk in TBST solution. This systematic approach allowed for the quantification of protein expression levels involved in the KRAS pathway inhibition following treatment with C14-PEI nanoplexes, providing insights into their therapeutic potential.^36^

### Wound healing assay

The μ-Dish with culture-insert 2 well (ibidi, Germany) was utilized for conducting a wound healing assay.^37^ Initially, 10 000 A549 cells suspended in 70 μL of RPMI-1640 media were added to each well and allowed to incubate at 37 °C with 5% CO_2_ for a minimum of 24 h to achieve a confluent cell layer. Following incubation, the insert was carefully removed using sterile tweezers, and the cell layer was washed twice with PBS to eliminate any cell debris and non-adherent cells. Subsequently, the μ-Dish was filled with 2 mL of fresh complete medium containing either PEG-PLE/C14-PEI nanoparticles or Lipofectamine 2000, as per experimental requirements. The cells were maintained in the incubator at 37 °C with 5% CO_2_ throughout the experiment, and images were captured at 0, 4, 8, and 24 h using an EVOS microscope (Thermo Fisher, Germany). The area of the wound gap was quantified and analyzed using ImageJ software, providing insights into the migration and healing dynamics of the A549 cell monolayer in response to the treatments administered.

### Cell apoptosis

Annexin V and propidium iodide (PI) staining allowed for the quantification of apoptotic and necrotic cells, providing insights into the cellular response toward PEG-PLE/C14-PEI nanoparticle transfection.^38^ A total of 1 × 10^5^ cells per well were initially seeded onto a 6-well plate in RPMI-1640 complete medium and transfected with PEG-PLE/C14-PEI nanoparticles. Following a 48-hour incubation at 37 °C with 5% CO_2_, the cells were washed twice with cold PBS and resuspended in Annexin V Binding Buffer at a concentration of 1 × 10^6^ cells per mL. Subsequently, 100 μL of the cell suspension was mixed with 10 μL of Annexin V-AF488 (Thermo Fisher) and 1 μL of PI (BD Biosciences), and the mixture was incubated for 15 min at room temperature in the dark. After incubation, 400 μL of Annexin V Binding buffer was added to each tube to halt the reaction. Fluorescence signals from Annexin V-AF488 and PI staining were measured using the Attune NxT flow cytometry (Thermo Fisher, Germany), and the data were analyzed using FlowJo software.

### Statistics

Unless otherwise specified, all results are presented as the mean value ± standard deviation (SD) based on triplicate experiments (*n* = 3). Statistical significance was investigated using one-way ANOVA or two-way ANOVA. All statistical analysis was performed using GraphPad Prism software (GraphPad Software, USA).

## Results

### Nanoparticle preparation

**Scheme 1:**
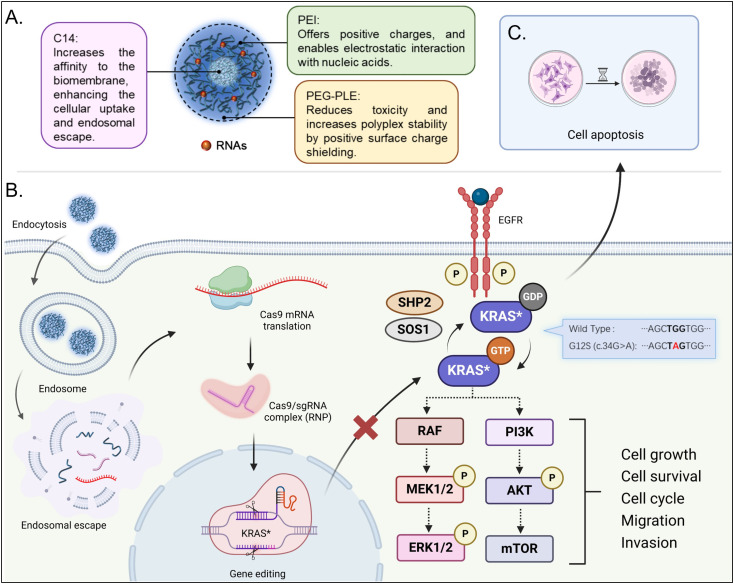
The strategy of PEG-PLE/C14-PEI for co-delivery of Cas9 mRNA and sgRNA targeting KRAS G12S. A. PEG-PLE/C14-PEI nanoplexes and the contributions of each component; B. Cas9 mRNA and sgRNA are released from nanoplexes and form CRISPR RNPs after mRNA translation to mediate gene editing in cell nuclear, leading to the downregulation of downstream signals; C. The deletion of KRAS G12S results lung cancer cell apoptosis. Created in BioRender (2024).

Although cationic polymers have been widely used for CRISPR Cas9 delivery, their performance in mRNA and sgRNA delivery systems is still unsatisfactory.^39^ Literature suggests that coating with anionic polymers can shield positive charges and enhance the properties of nanoparticles, making PEG-PLE a potential candidate for this purpose.^25,29,31^ We prepared nanoplexes with lipid-modified polymer (C14-PEI) and mRNA at a w/w ratio of 8 in 10 mM HEPES buffer at pH 7.4 and coated these nanoplexes with PEG-PLE at various mass ratios relative to C14-PEI (Scheme 1).

**Fig. 1:**
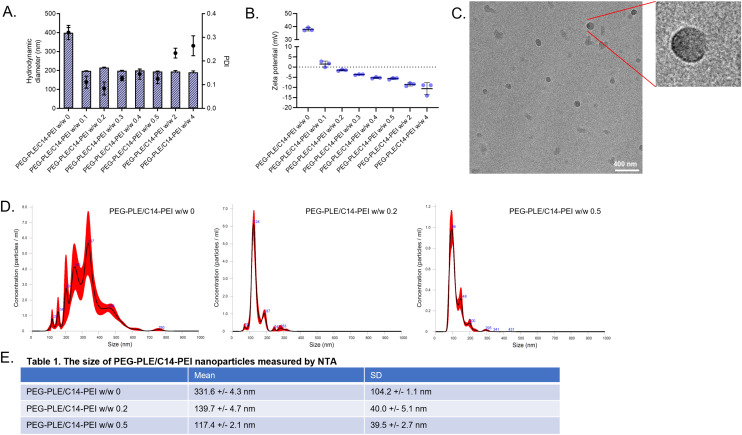
Characterization of PEG-PLE/C14-PEI. (A) Hydrodynamic diameters (bars) and polydispersity indices (PDI, dots) of nanoplexes (*n* = 3); (B) zeta potentials of nanoplexes (*n* = 3); (C) Cryo-EM image of PEG-PLE/C14-PEI; (D) size distributions of PEG-PLE/C14-PEI measured by NTA; (E) the table of NTA results.

The hydrodynamic diameter, PDI, and ζ potential of the nanoparticles were measured using DLS and LDA, respectively. As shown in Fig. 1A, without PEG-PLE coating, the C14-PEI nanoplexes with mRNA at a w/w ratio of 8 had a size of approximately 400 nm and a PDI of 0.3. In contrast, PEG-PLE/C14-PEI nanoparticles exhibited sizes ranging from 100 to 200 nm for PEG-PLE to C14-PEI ratios of 0.1 to 0.5, with an average PDI of 0.1. Even with a minimal amount of PEG-PLE at a polymer w/w ratio of 0.1, the nanoparticles maintained a compact size and effective charge shielding. Theoretically, at a mass ratio of 2.6, PEG-PLE is expected to neutralize the positive charges of C14-PEI. However, neutral charge was experimentally found around PEG-PLE/C14-PEI w/w 0.1, while nanoparticles at w/w 2 exhibited strong negative zeta potentials, indicating an excess of anions in the system (Fig. 1B). This can be attributed to the random polymer modification process, which may lead to errors in the N/P calculation.

C14-PEI nanoplexes had a zeta potential of approximately 40 mV. Increasing PEG-PLE content reduced the zeta potential, reaching near-neutral values (0 mV) at a polymer w/w ratio of 1.5 and transitioning to negative values at polymer w/w ratios of 2 and above. As the ratio increased, the zeta potential became more negative, ranging from −1.0 to −14 mV (Fig. 1B). Nanoparticles with zeta potentials between +10 mV and −10 mV are approximately neutral and often considered ideal, as this range provides sufficient electrostatic repulsion to prevent agglomeration, maintain stability, and reduce interactions with negatively charged cell membranes, thus reducing potential cytotoxicity and immune responses.^40,41^ Based on these properties, PEG-PLE/C14-PEI nanoparticles with polymer w/w ratios of 0.2 and 0.5 were selected for further experiments.

To further verify the size of PEG-PLE/C14-PEI nanoparticles, we employed Nanoparticle Tracking Analysis (NTA) using a Malvern NanoSight Pro system. As shown in Fig. 1D and E, the C14-PEI nanoparticles had an average size of 331.6 ± 4.3 nm with a standard deviation (SD) of 104.2 ± 1.1 nm. In comparison, PEG-PLE/C14-PEI nanoparticles with w/w ratios of 0.2 and 0.5 measured 139.7 ± 4.7 nm (SD: 40.0 ± 5.1 nm) and 117.4 ± 2.1 nm (SD: 39.5 ± 2.7 nm), respectively, indicating that the presence of PEG-PLE resulted in smaller and more uniformly sized nanoparticles. The sizes obtained from NTA were smaller than those measured by DLS. This discrepancy arises from the different methodologies employed by the two techniques. NTA tracks the trajectories of individual particles under a microscope, correlating their movement to size. In contrast, DLS measures the intensity fluctuations of scattered light, which reflects particle diffusion.^42^ Larger particles can dominate the scattered light signal in DLS, potentially dominating over smaller particles and leading to less accurate size determination.^43,44^ This explains the broader size range and lower reproducibility observed with DLS for the C14-PEI formulation.

Cryo-electron microscopy (Cryo-EM) was used to confirm the micelle structure of C14-PEI and to examine the morphology of PEG-PLE/C14-PEI nanoparticles. As shown in Fig. 1C, PEG-PLE/C14-PEI nanoplexes displayed a spherical shape with a more compact surface compared with C14-PEI nanoplexes (Fig. S2†). A distinct shadow on the surface indicates the presence of PEG-PLE coating. Fig. 1C displays the geometric particle sizes of PEG-PLE/C14-PEI at w/w 0.2, which align with the NTA results, showing sizes around 130 nm. This further confirmed that, for the highly dispersed particle suspensions with large sizes, NTA can offer a higher resolution of peaks and more precise particle size distribution compared with DLS.

### Cytotoxicity

**Fig. 2:**
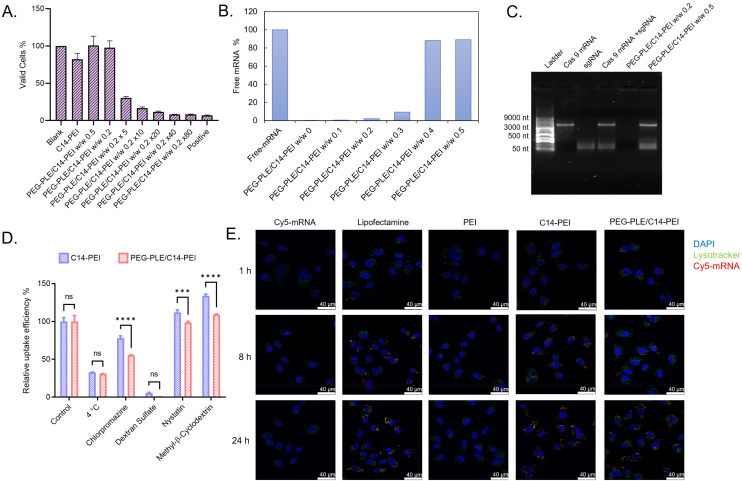
The assessment of RNA delivery with PEG-PEL/C14-PEI. (A) The cytotoxicity tests of PEG-PLE/C14-PEI by CCK-8, results are showed with viable cells after 24 h transfection in A549 cells (*n* = 3). (B) SYBR Gold assay to assess the encapsulation, results are showed as percent of free mRNA (*n* = 3); (C) agarose gel shows the co-encapsulation of Cas9 mRNA and sgRNA with different formulations; (D) the inhibition of celluler uptake pathways with C14-PEI and PEG-PLE/C14-PEI (***P ≤ 0.0002, ****P ≤ 0.0001); (E) endosomal entrapment of different formulations imaged *via* CLSM.

Cationic carriers facilitate the delivery of nucleic acids by interacting with cell membranes through electrostatic forces. However, an excess of cationic materials can disrupt the dynamic cell membrane and cause significant cytotoxicity.^20,45^ By neutralizing the cationic charges, negatively charged polymers can offer improved biocompatibility compared to traditional cationic delivery systems.^25^ To evaluate cytotoxicity, we used the CCK-8 assay, which measures the intracellular reduction of tetrazolium salt (WST-8) to produce an orange water-soluble formazan dye. This reaction, facilitated by the electron carrier 1-Methoxy PMS, produces a dye whose absorbance correlates linearly with the number of metabolically active cells, providing a direct measure of cytotoxicity. We assessed PEG-PLE/C14-PEI nanoplexes at polymer w/w ratios of 0.2 and 0.5 across various concentrations (1×, 5×, 10×, 20×, 40× increase based on the w/w 0.2 ratio) and compared them with C14-PEI complexes and a lysis buffer control. As shown in Fig. 2A, compared with PEI-C14 which illustrated 82% valid cells, PEG-PLE/C14-PEI nanoplexes showed less toxicity at w/w 0.2 and w/w 0.5 (98% and 101% valid cells respectively). This indicates that nanoplex biosafety and biocompatibility are improved with the shielding of the positive charges. However, at higher concentrations, cell viability decreased significantly. Cytotoxicity began to increase noticeably at a 5-fold concentration, resulting in 75% cell death. At a 40-fold increase, cell death approached 98%, comparable to the lysis buffer positive control.

### Encapsulation

Encapsulation efficiency is crucial for evaluating mRNA delivery systems due to mRNA's inherent instability and susceptibility to degradation by nucleases.^46^ To assess encapsulation, we used SYBR Gold, a fluorescent dye that binds to free nucleic acids and fluoresces upon excitation at 495 nm.^33^ This method leverages the interaction between cationic polymers and the negatively charged phosphate groups of mRNAs, which promotes mRNA encapsulation within nanoparticles through charge complexation. As a result, the fluorescence intensity of SYBR Gold decreases, allowing for the quantification of free mRNA in nanoparticle suspensions. Fig. 2B shows the results using C14-PEI as a control, with free mRNA set at 100%. The percentage of free mRNA increased as the amount of PEG-PLE increased. At PEG-PLE to C14-PEI mass ratios below w/w 0.3, mRNA encapsulation exceeded 90%. Specifically, at ratios of w/w 0.1 and w/w 0.2, the encapsulation efficiencies were 99% and 98%, respectively, comparable to the C14-PEI group. However, at a mass ratio of w/w 0.4, mRNA release began, with only 12% encapsulated. At w/w 0.5, the free mRNA increased to approximately 90%, indicating poor mRNA condensation. This reduced efficiency can be attributed to the competition with the negatively charged poly(l-glutamic acid) (PGA), which competes with mRNA for binding the positively charged PEI. PEG-PLE contains l-glutamic acid sodium salt, which is ionized into sodium and glutamate ions when dissolved in water, resulting in negative charges in the polymer. Literature suggests that the stability of polyplexes can be compromised by competing anions.^47^ Anionic PEG-PLE will compete with mRNAs for cationic C14-PEI binding position. Hence, the mRNA binding will be reduced at a high concentration of PEG-PLE. While the positive charge of polymers facilitates mRNA encapsulation through electrostatic interactions, strong polymer–mRNA binding can also impede mRNA release.^48^ In this study, the nanoparticle formulations with intermediate negative-to-positive polymer w/w ratio demonstrated a balanced interaction between polymers and mRNA, allowing sufficient encapsulation and mRNA release in the presence of competing molecules at the same time.

Additionally, co-encapsulation of Cas9 mRNA and sgRNA was evaluated using electrophoretic mobility shift assays (EMSA). In this assay, negatively charged free RNA migrates through the agarose gel, while encapsulated RNA remains in the wells due to the larger size of the nanoparticles relative to the gel mesh size. Fig. 2C illustrates that free Cas9 mRNA and sgRNA are present as bands at 4500 nt and 100 nt, respectively (lanes 2 and 3). Lane 4 shows the bands of a mixture of free Cas9 mRNA and sgRNA. In contrast, lane 5, containing PEG-PLE/C14-PEI at polymer w/w 0.2, displays no bands on the gel but a bright signal around the wells, indicating encapsulation of both Cas9 mRNA and sgRNA within nanoparticles. Conversely, lane 6 shows two RNA bands for PEG-PLE/C14-PEI at polymer w/w 0.5, suggesting that neither Cas9 mRNA nor sgRNA was encapsulated, consistent with the results from the SYBR Gold assay.

### Uptake pathway

The route of cellular uptake plays a crucial role in determining the intracellular processing and transfection efficiency of delivery systems. For instance, it has been established that lipoplexes are predominantly internalized *via* clathrin-mediated endocytosis, whereas polyplexes utilize both clathrin-mediated and caveolae-mediated endocytosis.^49,50^ However, the caveolae-dependent route appears to lead to more successful transfection,^49^ as polyplexes and their payloads often undergo lysosomal degradation following clathrin-mediated entry. Furthermore, the internalization of nanoparticles is generally considered to be an energy-dependent endocytosis mechanism.^51,52^ To elucidate the uptake pathway of PEG-PLE/C14-PEI nanoparticles, we performed a cellular uptake experiment comparing PEG-PLE/C14-PEI with C14-PEI nanoplexes. Cells were incubated with various chemical uptake inhibitors, including nystatin, dextran sulfate, chlorpromazine, and methyl-β-cyclodextrin, along with a low-temperature (4 °C) inhibition group, prior to transfection. The samples were then processed and analyzed using flow cytometry. Each inhibitor targets different pathways: nystatin inhibits caveolae and lipid raft-mediated endocytosis by depleting cholesterol from the cell membrane;^53^ dextran sulfate inhibits scavenger receptor-mediated endocytosis;^52^ chlorpromazine disrupts clathrin-coated pit formation by causing clathrin to translocate from the plasma membrane to intracellular vesicles;^54^ and methyl-β-cyclodextrin inhibits cholesterol-dependent endocytosis by depleting membrane cholesterol.^55^ The cellular uptake data, expressed as a percentage of mean fluorescence intensity (MFI) relative to uninhibited samples, provided insight into the primary uptake pathways of these nanoparticles.

The low-temperature group and dextran sulfate treatments significantly inhibited nanoplex uptake, reducing cellular uptake by approximately 70% and 90%, respectively, for both C14-PEI and PEG-PLE/C14-PEI formulations (Fig. 2D). This indicates that both formulations predominantly rely on energy-dependent endocytosis and scavenger receptor-mediated pathways. Notably, there was no significant difference in uptake between the two formulations under these conditions, suggesting that energy-dependent endocytosis and scavenger receptor-mediated internalization are equally important for both nanoparticle types. Given the strong inhibitory effects of dextran sulfate, it is likely that polyplexes with higher lipid content, such as PEG-PLE/C14-PEI, share similarities with lipoplexes regarding their uptake route. Indeed, lipid nanoparticles are often internalized *via* scavenger receptor-mediated uptake.^56,57^ The remaining uptake after treatment with nystatin, chlorpromazine, and methyl-β-cyclodextrin was 112.09%, 77.89%, and 133.97% for C14-PEI, and 98.74%, 55.65%, and 109.20% for PEG-PLE/C14-PEI, respectively, compared to uninhibited conditions (Fig. 2D). These results suggest that neither formulation primarily relies on caveolae-mediated or cholesterol-dependent endocytosis. Instead, they are only partially dependent on clathrin-mediated endocytosis. Recent studies have shown that while lipoplexes are taken up *via* clathrin-mediated endocytosis, PEI polyplexes lose transfection efficiency if caveolae-mediated endocytosis is blocked.^49^ Furthermore, amphiphilic polyplexes have been found to depend on both clathrin-mediated endocytosis and fusogenic uptake mechanisms. These findings collectively suggest that PEG-PLE/C14-PEI nanoparticles utilize a complex uptake mechanism, with a potential preference for pathways that avoid lysosomal degradation, thereby enhancing transfection efficiency.^33^

### Endosomal entrapment

Through our cellular uptake experiments, we confirmed that the internalization of nanoparticles *via* the endocytic pathway is consistent with previous reports.^58^ During this process, nanoparticles are typically trapped within endosomes and eventually degraded by lysosomal enzymes. To ensure effective biological effects, it is essential for these nanoparticles to escape from lysosomes and deliver their therapeutic payloads to the cytosol.^59^ To visualize endosomal entrapment and the subsequent escape of nanoparticles within cells, we transfected A549 cells with Cy-5 labeled mRNA. We used LysoTracker Green DND-26, a fluorescent dye that specifically stains acidic compartments such as lysosomes, and DAPI to stain the cell nuclei. Confocal laser scanning microscopy (CLSM) was employed to co-locate the mRNA with the lysosomes, allowing for detailed imaging of the intracellular distribution and release dynamics of the nanoparticles. The study compared the C14-PEI and PEG-PLE/C14-PEI formulations, with PEI and Lipofectamine 2000 serving as controls. In the microscopy images (Fig. 2E), the blue areas represent cell nuclei stained with DAPI, red dots indicate the presence of Cy-5 labeled mRNA, green regions correspond to lysosomes stained by LysoTracker, while yellow dots signify mRNA that is co-localized within lysosomes. In the control groups of free mRNA and PEI-transfected cells, there were no red dots and only a few green dots, indicating that the mRNA did not successfully transfer into the cytoplasm. In contrast, the Lipofectamine 2000 group exhibited a punctate distribution of Cy-5 labeled mRNA (red) as early as 1 hour after transfection, along with the formation of acidic lysosomes (green dots). The signal intensity increased over time, with maximum mRNA uptake observed at 24 h.

For the C14-PEI and PEG-PLE/C14-PEI formulations, red dots were clearly visible on the cell membrane surfaces within 1 hour, indicating the initiation of internalization. After 8 h, numerous acidic lysosomes had formed, and significant co-localization (yellow dots) with the mRNA presented. Maximum uptake was observed after 24 h for both formulations. While yellow dots persisted after 24 h, signifying partial entrapment within endosomes, a substantial portion of the mRNA managed to escape and disperse into the cytoplasm. Notably, compared to C14-PEI, the PEG-PLE/C14-PEI formulation displayed more red dots and fewer yellow dots after 24 h, suggesting a higher efficiency in endosomal escape. One commonly proposed mechanism for the endosomal escape of polyplexes is the “proton sponge effect”.^60^ According to this hypothesis, once inside the acidifying environment of endosomes or lysosomes, materials containing amine groups can sequester endosomal protons, thereby slowing the pH drop. As a result, cells pump additional protons into the endosomes to reach the target pH, leading to an influx of counterions and an increase in osmotic pressure within the endosomes. This heightened pressure can cause the endosomal membrane to rupture, facilitating the escape of the delivery system into the cytoplasm. However, emerging evidence suggests that the process of polyplex-mediated endosomal escape is more complex than just the proton sponge effect. For instance, it has been shown that introducing PEG to create long-circulating nanoparticles can inhibit endosomal escape.^60^ Moreover, studies have highlighted that the fusion of hydrophobic nanoparticles with lysosomal or endosomal membranes occurs through a combination of hydrophobic interactions, pH-triggered responses, and membrane destabilization, enabling the release of cargo into the cytoplasm.^59,61^ Hydrophobic or amphiphilic nanoparticles often interact more readily with these membranes, facilitating fusion by promoting closer contact between the hydrophobic parts of the particles and the membrane lipids.^62^ This interaction, driven by direct electrostatic forces with negatively charged membrane components or the insertion of hydrophobic domains, can destabilize the membrane, allowing cargo or nanoparticles to escape. Additionally, certain nanoparticles remain stable at neutral pH but become more hydrophobic or undergo charge alterations in acidic conditions, further promoting membrane fusion and escape.^63^ Additionally, a report by Galliani *et al.* demonstrated that drugs delivered *via* anionic poly(lactic-*co*-glycolic) acid (PLGA) nanoparticles exhibited a lower degree of co-localization with lysosomes after 2 h of incubation, which was attributed to a burst release mechanism.^64^ These findings highlight the need for further research to fully understand the mechanisms behind endosomal escape and improve the design of nanoparticle-based delivery systems.^65^

### Co-localization of Cas9 mRNA and sgRNA

Co-delivering Cas9 mRNA and sgRNA presents a significant challenge, primarily due to the risk of sgRNA degradation before it can effectively pair with the Cas9 protein. To ensure sufficient sgRNA is available for the formation of ribonucleoprotein complexes (RNPs), strategies such as increasing sgRNA quantity and enhancing its stability through modification have been employed.^17,66^ To better understand the intracellular distribution, kinetics, and behavior of Cas9 mRNA and sgRNA, we developed a fluorescence labeling-based method for tracking these molecules using confocal microscopy. Cas9 mRNA was synthesized *via in vitro* transcription using Cy5-UTP, while sgRNA, sourced from Sigma-Aldrich, was labeled with pCp-AF488 (Jena Bioscience, Germany). These labeled RNAs were co-delivered to A549 cells using PEG-PLE/C14-PEI at polymer weight ratios (w/w) of 0.2 and 0.5, with C14-PEI and Lipofectamine 2000 serving as comparison controls. Fluorescence images were captured at various time points: 1, 4, 8, 24, 32, 48, and 72 h, allowing us to estimate the relative duration and degradation of the RNAs by measuring fluorescence intensity.

**Fig. 3:**
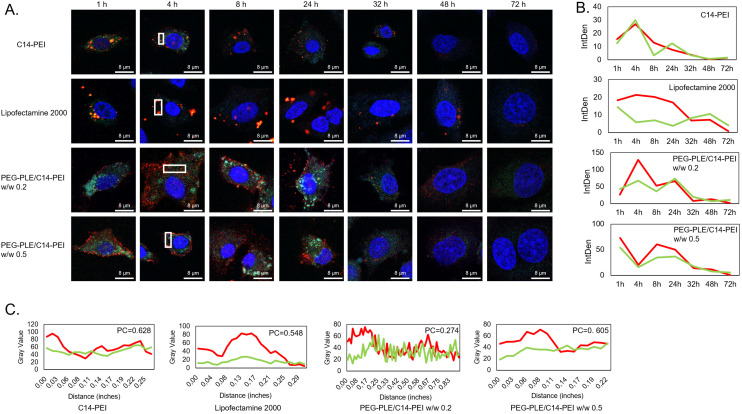
Co-localization of Cas9 mRNA and sgRNA. (A) The imges of different formulations in 72 h post transfection, captured by CLSM; (B) integrated density of Cy5-mRNA and AF88-sgRNA related to Figure A in 72 h, density was assessed by imageJ; (C) plot profile of ROIs of Figure A at 4 h post transfection, co-localization is analyzed by Pearson's correlation coefficient (PC).

As illustrated in Fig. 3A, C14-PEI, Lipofectamine 2000, and PEG-PLE/C14-PEI demonstrated distinct behaviors and distribution patterns over 72 h. Both C14-PEI and Lipofectamine 2000 showed high levels of co-localization of Cas9 mRNA and sgRNA, with Lipofectamine 2000, in particular, forming larger, more enriched complexes. This is likely due to Lipofectamine 2000s mechanism of action, where nucleic acids are encapsulated in lipoplexes, facilitating their uptake *via* clathrin-mediated endocytosis and resulting in enriched vesicular distribution. In contrast, C14-PEI quickly released the RNAs, leading to a more homogeneous distribution within cytoplasmic compartments. However, as Fig. 3B shows, this formulation provided less protection for the RNAs, resulting in faster degradation and a shorter intracellular lifespan. Interestingly, the PEG-PLE/C14-PEI formulation exhibited a delayed yet more efficient RNA distribution. In Fig. 3B, the signal accumulation of PEG-PLE/C14-PEI formulation kept a high level after 24 h, with Cas9 mRNA and sgRNA displaying distinct distribution patterns and lower co-localization in Fig. 3C. Cy5-labeled Cas9 mRNA initially concentrated on the cell surface before gradually dispersing into the cytoplasm, while AF488-labeled sgRNA quickly localized to the cytoplasm and subsequently migrated into the nucleus over time (Fig. 3A). This distribution aligns with the expected mechanism, where Cas9 mRNA is translated into Cas9 protein in the cytoplasm, which then interacts with sgRNA within the nucleus.^3^

In summary, the PEG-PLE/C14-PEI formulation not only facilitated a more efficient distribution of Cas9 mRNA and sgRNA but also extended their intracellular persistence. This prolonged presence resulted in higher expression levels and greater gene-editing efficiency compared to C14-PEI.

### Luciferase mRNA expression

**Fig. 4:**
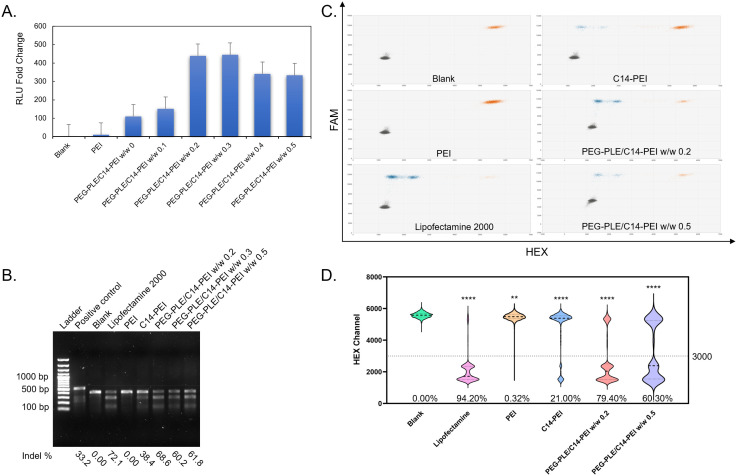
Fluc mRNA expression and gene editing efficiency. (A) RLU fold change after PEG-PLE/C14-PEI transfection 24 h, nomalized by the blank; (B) T7EI cleavage tests in agarose gel; edited efficiency is labeled below the image; (C) droplet distribution of ddPCR, *X*-axis is HEX channel, *Y*-axis is FAM channel, gray dots designate the FAM-negative/HEX-negative group, orange dots represent the FAM-positive/HEX-positive group, blue dots are the FAM-positive/HEX-negative group; (D) Violin plots of HEX channel (excludes FAM negative droplets) of ddPCR (**P ≤ 0.0021, ****P ≤ 0.0001); intensity at 3000 is set as threshold, and edited efficiency is labeled below the plots.

To evaluate the mRNA expression efficiency of the PEG-PLE/C14-PEI formulation, luciferase mRNA (Fluc mRNA) was transfected into A549 cells at various mass ratios of PEG-PLE to C14-PEI: 0, 0.1, 0.2, 0.3, 0.4, and 0.5. For comparison, PEI and C14-PEI formulations were used as controls. Following transfection, relative luminescence units (RLU) were measured with a plate reader (TECAN). As shown in Fig. 4A, all PEG-PLE/C14-PEI groups successfully induced luciferase expression. Notably, the inclusion of PEG-PLE in the C14-PEI nanoparticles significantly enhanced luciferase expression levels. The highest RLU was observed in the 0.3 w/w PEG-PLE group, representing a 385-fold increase compared to the blank control. Interestingly, mRNA expression did not increase linearly with the amount of PEG-PLE. At higher PEG-PLE ratios, the luciferase signals decreased, with the 0.5 w/w group showing around a 300-fold increase in RLU. The enhanced mRNA expression observed with the PEG-PLE/C14-PEI formulation is likely due to improved endosomal escape, as evidenced by the expression profile across different PEG-PLE ratios, which aligns with encapsulation test results. In conclusion, the PEG-PLE/C14-PEI formulations demonstrated more efficient endosomal escape and higher mRNA expression compared to the C14-PEI formulation.

### T7EI assay

To further assess the capability of the PEG-PLE/C14-PEI formulation to co-deliver Cas9 mRNA and sgRNA and facilitate gene editing, we transfected A549 cells, a cell line known to harbor *KRAS* G12S mutations,^67^ with Cas9 mRNA and sgRNA specifically targeting the *KRAS* G12S allele. The gene editing efficiency was then evaluated using PEG-PLE/C14-PEI nanoparticles. As described previously, non-homologous end joining (NHEJ) is the primary mechanism for CRISPR-Cas9-mediated gene knockout, often resulting in insertions and/or deletions (indels) in the DNA strand.^68^ T7 Endonuclease I, a structure-selective enzyme, recognizes these indel sites on the DNA sequence and cleaves them into two fragments.^69^ The resulting digestion products can be visualized and analyzed through agarose gel electrophoresis. C14-PEI and PEG-PLE/C14-PEI formulations at polymer weight ratios (w/w) of 0.2 and 0.5 were transfected into A549 cells for 48 h. PEI and Lipofectamine 2000 were used as controls. As shown in the gel imaging (Fig. 4B), samples treated with C14-PEI and PEG-PLE/C14-PEI nanoparticles demonstrated efficient gene editing after 48 h. The blank control, which received no treatment, showed only a single band corresponding to the target sequence, indicating no editing. The PEI control exhibited a similar result, confirming the absence of gene editing, likely due to the inability of mRNA to enter the cells, as corroborated by CLSM. In contrast, the Lipofectamine 2000-treated sample displayed both the original band and two cleaved bands, indicating a successful gene editing event with an indel percentage of 72.1%. This highlights the necessity of an appropriate delivery system for effective gene editing. Similarly, C14-PEI and PEG-PLE/C14-PEI at w/w ratios of 0.2 and 0.5 achieved average indel rates of 38.4%, 68.6%, and 60.2%, respectively. Notably, the PEG-PLE/C14-PEI formulation demonstrated higher gene editing efficiency than the C14-PEI group, consistent with its superior endosomal escape and increased luciferase expression observed in earlier experiments.

### Droplet digital PCR

The T7 Endonuclease I (T7EI) assay, while useful for detecting indels, is semi-quantitative, has limited sensitivity, and is prone to false positives. It also suffers from high background signals when sequence polymorphisms are present.^69^ To overcome these limitations, we employed droplet digital PCR (ddPCR) to more accurately assess the deletion of *KRAS* G12S alleles in A549 cells. In ddPCR, two specific probes within a single amplicon are used to detect NHEJ-mediated events.^34,70^ The first probe, labeled with FAM, serves as a reference and is located away from the mutagenesis site, counting all genomic copies of the target. The second probe, labeled with HEX, is positioned at the site of nuclease-induced cuts or nicks in the DNA. If NHEJ occurs, the HEX probe loses its binding site, resulting in the loss of the HEX signal, leaving only the FAM signal from the reference probe. To perform the ddPCR assay, genomic DNA was isolated from transfected cells 48 h post-transfection. The DNA was then subjected to droplet generation, PCR amplification, and fluorescence analysis.

As shown in Fig. 4C, droplets that were positive for both FAM and HEX (orange group) represent unedited DNA copies, while droplets positive for FAM but negative for HEX (blue group) represent edited DNA copies. Consistent with the T7EI assay results, no edited events were detected in the blank and PEI control groups. However, in the Lipofectamine 2000 group, 3017 positive droplets were observed, compared to 573 in the C14-PEI group, and 1768 and 617 edited events in the PEG-PLE/C14-PEI groups with w/w ratios of 0.2 and 0.5, respectively. Subsequently, we calculated the percentage of edited gene copies among the total events. As shown in Fig. 4D, the gene editing efficiency of Lipofectamine 2000 reached 94.2%, while C14-PEI achieved 21%. Notably, the PEG-PLE/C14-PEI formulation outperformed C14-PEI, with editing efficiencies of 79.4% and 60.3% in the w/w 0.2 and w/w 0.5 groups, respectively.

### Sanger sequencing

**Fig. 5:**
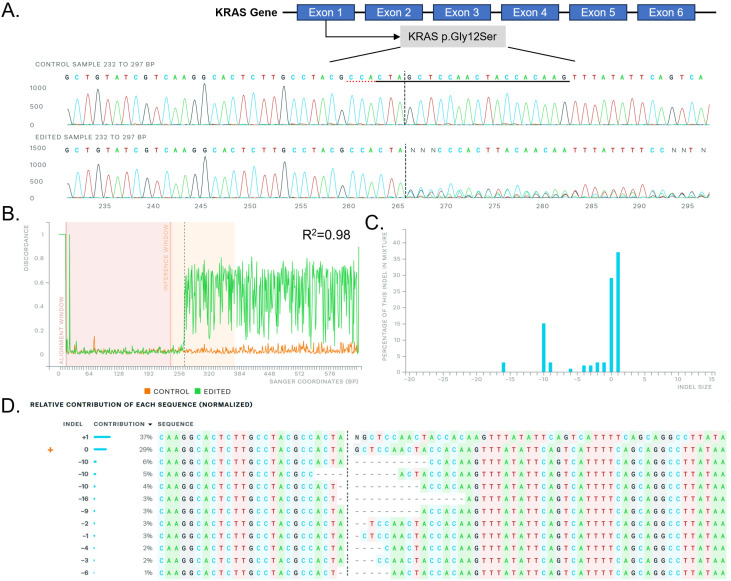
Sanger sequencing after PEG-PLE/C14-PEI w/w 0.2 treatment in A549 cells analyzed by the ICE CRISPR analysis tool. (A) KRAS exon map (up), and edited sequence (reverse strand, down) illustrate; (B) alignment of Sanger sequencing; (C) distribution of indel sizes; (D) contribution of each sequence after gene editing.

To further validate the gene editing efficacy of the PEG-PLE/C14-PEI formulation, we performed Sanger sequencing on the PCR products from PEG-PLE/C14-PEI treated A549 cells. The sequencing data was analyzed using the ICE CRISPR analysis tool,^35^ confirming that the *KRAS* G12S allele had been successfully edited by the PEG-PLE/C14-PEI system (Fig. 5A and D). Indels were detected around the protospacer adjacent motif (PAM) sequence (TGG) in the DNA backbone, indicating successful gene editing. The *KRAS* G12S editing efficiency showed a strong correlation (*R*^2^ = 0.98) based on sequence alignment (Fig. 5B). Overall, the analysis revealed that 69% of the sequences contained indels of varying sizes, while 29% exhibited base alterations. Among the detected indels, a 1 bp insertion was the most common, accounting for 37% of the total, which is consistent with findings by Gao and colleagues.^67^ This was followed by a −10 bp deletion (15%) and other indels (17%), aligning with the expected outcomes of NHEJ-mediated knockouts. The presence of these deletions can induce frameshift mutations within the *KRAS* gene, potentially leading to the functional inactivation of the mutant KRAS protein. The indels detected near the PAM sequence confirm the precision and efficiency of the PEG-PLE/C14-PEI delivery system in targeting *KRAS* G12S alleles, highlighting its potential for effective gene editing.

### Western blot

**Fig. 6:**
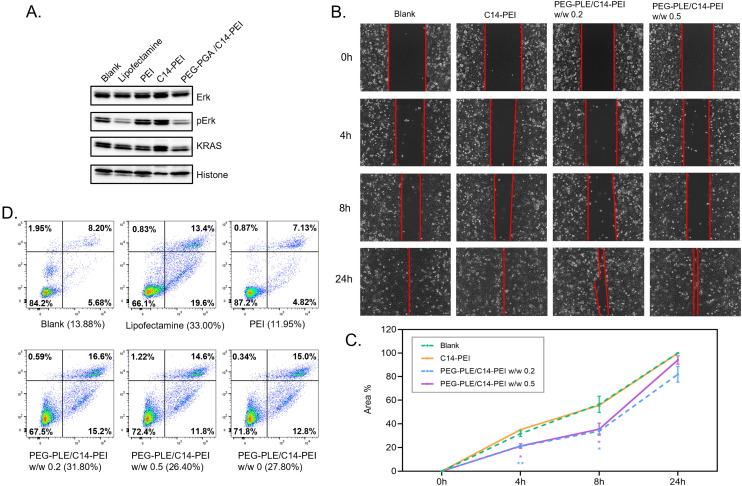
Cell capability assessment after the transfection of PEG-PLE/C14-PEI nanoplexes. (A) Western blot after 48 h transfection in A549 cells; (B) typical images of A549 cells in the wound healing assay 24 h after transfection, red lines indicate the front of migrating cells; (C) the line graphs depict the average percentages with SD (*n* = 3) of covered area in wound healing assay (*P ≤ 0.0332, **P ≤ 0.021, reflecting significance against C14-PEI); (D) cell apoptosis after 48 h transfection in A549 cells, *x*-axis shows intensity of Annexin V-AF488, and *y*-axis shows intensity of PI.

KRAS plays a crucial role in activating downstream effector molecules, including those in the MAPK and AKT-mTOR signaling pathways, which are essential for cell proliferation and survival (Scheme 1B).^71^ Therefore, assessing the protein levels in these downstream pathways after gene editing is critical. We performed a western blot analysis to evaluate the expression of downstream proteins following the knockout of the *KRAS* G12S allele. Given that mutant *KRAS* leads to the continuous activation of downstream signaling, particularly resulting in the phosphorylation of ERK, we focused on analyzing both total ERK and phosphorylated ERK (pERK) levels. Lipofectamine 2000 and PEI were used as controls for comparison. In the experiment, 100 000 A549 cells were seeded in a 6-well plate 24 h before transfection with PEG-PLE/C14-PEI. After 48 h, total protein was extracted from the cells and analyzed by western blotting. As shown in Fig. 6A, the total ERK levels remained consistent across all groups, indicating that the overall expression of ERK was not affected by the treatments. However, a significant downregulation of pERK was observed in the groups treated with Lipofectamine 2000 and PEG-PLE/C14-PEI, suggesting effective inhibition of downstream signaling following *KRAS* G12S knockout. In contrast, the C14-PEI formulation did not mediate a similar downregulation of pERK, possibly due to functional compensation by the cells, where protein translation does not always correlate directly with gene editing efficiency.^72^ This could explain why the C14-PEI formulation was less effective in downregulating pERK despite successful gene editing.

### Cell migration

Activating mutations in *KRAS* lead to impaired GTP hydrolysis or enhanced nucleotide exchange, resulting in continuous downstream signaling that drives sustained cell proliferation. This signaling is closely related to the migration and invasion of cancer cells.^71,73^ Therefore, knocking out mutant *KRAS* is expected to inhibit cancer cell migration. To evaluate the impact of *KRAS* gene editing on cell migration, we conducted a wound healing assay using a cell culture dish with a 2-well insert from ibidi (Germany). A549 cells were seeded into the insert chambers 24 h before transfection. The insert was removed once the cells reached 100% confluence. Following nanoparticle transfection, cell migration was assessed by measuring the gap area between the two cell groups from time 0 h to 24 h using ImageJ (Table S1†). As shown in Fig. 6B and C, cells treated with the PEG-PLE/C14-PEI formulations exhibited significantly slower migration compared to the C14-PEI control group, which shows a similar profile with the blank control. After 4 h, the C14-PEI group had covered approximately 35% of the wound area, while the PEG-PLE/C14-PEI w/w 0.2 and w/w 0.5 groups covered around 21%. After 8 h, the PEG-PLE/C14-PEI w/w 0.2 and w/w 0.5 groups covered nearly 35%. By 24 h, the wound was completely closed in the C14-PEI and blank groups, whereas the PEG-PLE/C14-PEI w/w 0.5 group showed 94% wound coverage, and the PEG-PLE/C14-PEI w/w 0.2 showed a further inhibition with 82% coverage though no significance was observed at this point. Consistent with western blot findings, the PEG-PLE/C14-PEI formulations mediated a stronger impact on cell migration compared to the C14-PEI group, further demonstrating the superior efficacy in inhibiting tumor cell proliferation.

### Cell apoptosis

Cell apoptosis is a crucial indicator for evaluating the effectiveness of *KRAS* mutation excision following CRISPR-Cas9 treatment, as *KRAS* is integral to cell proliferation. To investigate whether *KRAS* mutant deletion induces apoptosis in cancer cells, we assessed the percentage of apoptotic cells using flow cytometry with an Annexin V-AF488/propidium iodide (PI) double-staining assay.^38^ The assay was conducted on A549 cells treated with PEG-PLE/C14-PEI formulations at w/w ratios of 0.2 and 0.5, with C14-PEI, Lipofectamine 2000, and PEI used as controls. The representative flow cytometry data are shown in Fig. 6D. After 48 h post-transfection, the Lipofectamine 2000 group exhibited the highest percentage of apoptotic cells at 33.00%. The C14-PEI group showed a slightly lower apoptosis rate at around 28.00%. In contrast, the PEI group demonstrated only 11.95% apoptosis, indicating minimal gene editing effects. The PEG-PLE/C14-PEI groups showed 31.8% and 26.4% apoptotic cells for the w/w 0.2 and w/w 0.5 formulations, respectively. These results reflect a significant increase in apoptosis compared to the blank and PEI groups. Notably, the PEG-PLE/C14-PEI w/w 0.2 formulation achieved apoptosis levels comparable to Lipofectamine 2000, demonstrating its effectiveness in inducing cell apoptosis through *KRAS* mutant excision.

## Conclusions

Polymeric nanocarriers have played a crucial role in delivering a wide variety of nucleic acids, including DNA,^74^ RNAs,^75,76^ and oligonucleotides.^77^ However, for co-delivery of Cas9 mRNA and sgRNA, the efficiency of each component must be carefully optimized to ensure effective gene editing due to the necessity of delivering two kinds of RNAs with different molecular sizes and structures.^24^ In this study, we explored the use of methoxy-poly(ethylene glycol)-*block*-poly(l-glutamic acid sodium salt) (PEG-*b*-PLE) to address these issues by shielding the positive charges of C14-PEI formulations, aiming to enhance the nanoparticles’ properties and delivery efficiency. We prepared PEG-PLE/C14-PEI nanoparticles by blending PEG-PLE into the RNA solution, varying the w/w ratios of PEG-PLE to C14-PEI from 0 to 4. Characterization through DLS and LDA revealed that PEG-PLE significantly reduced the nanoparticle size from approximately 330 nm to around 140 nm, as confirmed by NTA. The zeta potential also decreased from nearly 40 mV to a slight negative charge range of −1.0 mV to −14 mV. Among the formulations, PEG-PLE/C14-PEI at a polymer w/w ratio of 0.2 exhibited optimal properties, including low toxicity, high encapsulation efficiency, and effective mRNA delivery. Confocal microscopy imaging showed that PEG-PLE/C14-PEI efficiently escaped from endosomes and distributed Cas9 mRNA and sgRNA within cells. Uptake pathway inhibition tests indicated that PEG-PLE/C14-PEI internalization primarily relies on scavenger receptors and clathrin-mediated endocytosis. Notably, the PEG-PLE/C14-PEI w/w 0.2 formulation achieved the highest gene editing efficiency for *KRAS* G12S deletion in A549 cells, with 68.6% indels detected by T7EI and 79.4% edited signals observed by ddPCR. Sanger sequencing confirmed *KRAS* G12S deletion with 69% of indels and 29% of base alterations. Following *KRAS* G12S deletion, western blot analysis showed reduced levels of phosphorylated ERK, and approximately 32% of apoptotic cells were observed in PEG-PLE/C14-PEI w/w 0.2-treated cells. Additionally, cell migration was significantly decreased after treatment with the PEG-PLE/C14-PEI formulation. These findings demonstrate that PEG-PLE, as a negatively charged polymer, effectively enhances polycationic nanoplex properties, increases mRNA expression, and improves gene editing efficiency by providing surface adsorption and charge shielding. Especially, when the mass ratio between PEG-PLE and C14-PEI is 0.2, the formulation performed the most promising behavior and efficiency in characterization, encapsulation, mRNA expression, and gene editing. Future studies will determine *in vivo* gene editing in lung cancer.

## Author contributions

Siyu Chen: data curation, investigation, methodology, and writing – original draft. Simone P. Carneiro: methodology, project administration, supervision, and writing – review. Olivia M. Merkel: conceptualization, funding acquisition, supervision, and writing – review and editing.

## Data availability

All data generated or analysed during this study are included in the main manuscript and in the ESI.†

Raw data supporting all findings reported in this manuscript can be found at: https://syncandshare.lrz.de/getlink/fiUBhcHm25pCjPkpzGgrTL/Chapter%20II.

## Conflicts of interest

There are no conflicts to declare.

## Supplementary Material

BM-013-D4BM01290A-s001
