# Supplementary material for: Anionic polymer coating for enhanced delivery of Cas9 mRNA and sgRNA nanoplexes
Source: Biomater Sci. 2024 Dec 11;13(3):659–76. doi: 10.1039/d4bm01290a (PMC11650648; doi:10.1039/d4bm01290a)
Supplement: BM-013-D4BM01290A-s001 [file BM-013-D4BM01290A-s001.pdf]

Supplementary Information (SI) for Biomaterials Science.
This journal is © The Royal Society of Chemistry 2024

Supplementary Information

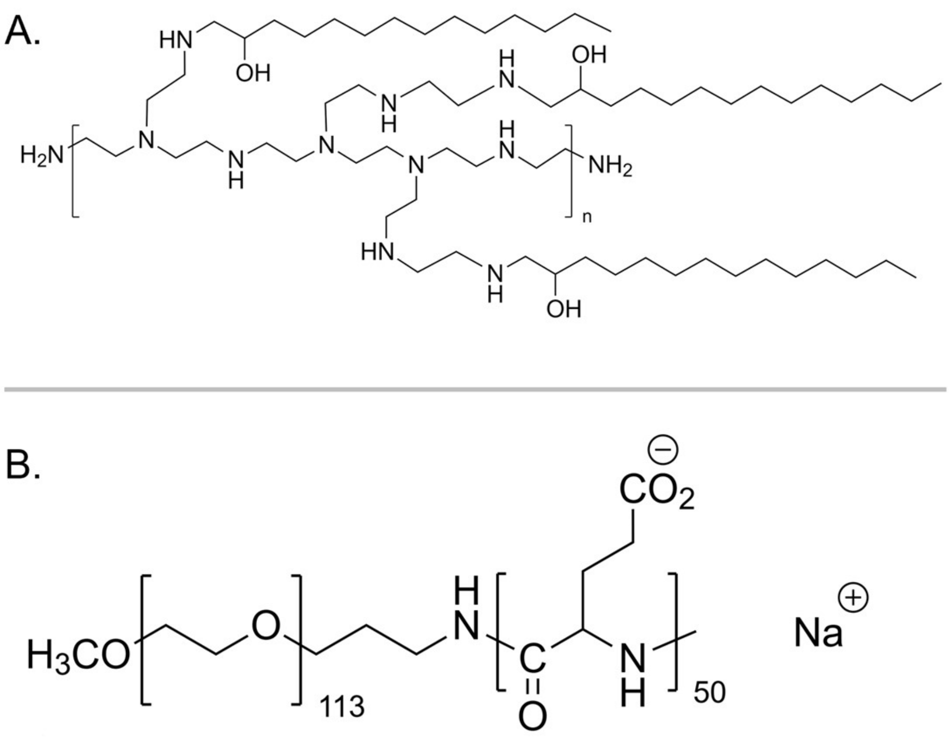

Figure S1. A. the chemical structures of C14-PEI; and B. PEG-PLE.

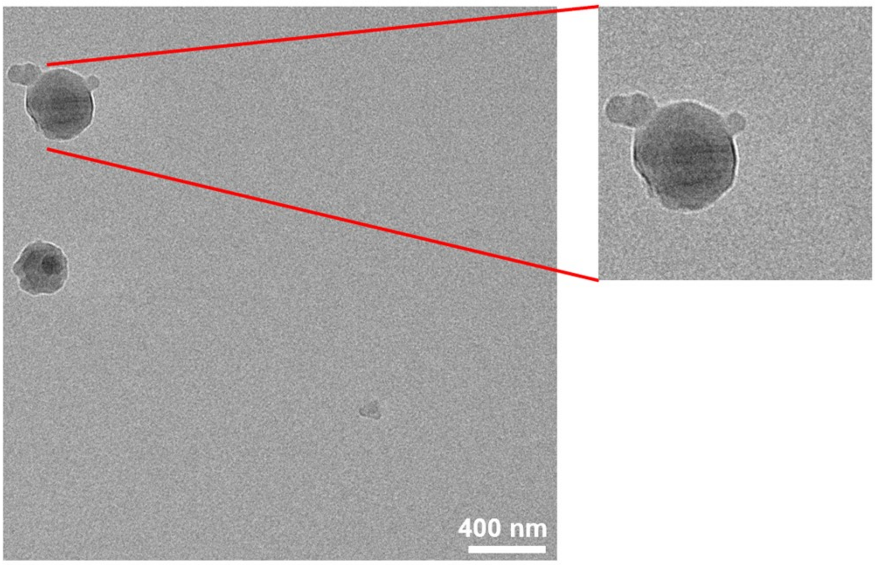

Figure S2. Cryo-EM image of C14-PEI nanoparticles.

Uncovered Area (Pixels)	0h			4h			8h			24h		
Blank	1312919	1309065	1301590	918533	857203	903438	598260	642215	466384	0	0	0
C14-PEI	1273331	1278102	1261105	822393	834535	816370	576807	553308	557377	0	0	4892
PEG-PLE/C14-PEI w/w 0.2	1467265	1165410	1168270	1162051	909283	930458	944096	761768	803898	251631	139939	293141
PEG-PLE/C14-PEI w/w 0.5	1135381	1170014	1116487	917249	899919	875391	794744	701866	708277	65434	111302	27251

Table S1. The uncovered area in cell migration tests.
